# Three-year outcomes of cystotomy for cystoid macular edema secondary to retinal vein occlusion

**DOI:** 10.1371/journal.pone.0332941

**Published:** 2025-09-22

**Authors:** Yasuyuki Sotani, Hisanori Imai, Hiroko Yamada, Akiko Miki, Makoto Nakamura

**Affiliations:** 1 Department of Surgery, Division of Ophthalmology, Kobe University Graduate School of Medicine, Kusunoki-cho, Chuo-ku Kobe, Japan; 2 Department of Ophthalmology, Kansai Medical University, Shin-machi, Hirakata, Japan; Yokohama City University, JAPAN

## Abstract

This retrospective observational study evaluated the three-year clinical outcomes of cystotomy for managing refractory cystoid macular edema (CME) secondary to retinal vein occlusion (RVO). A total of 23 eyes from 23 patients (10 males, 13 females) with CME secondary to RVO (RVO-ME) who underwent cystotomy at Kobe University Hospital between September 2014 and July 2021 were reviewed, with a minimum follow-up of 3 years. Clinical parameters such as age, sex, best-corrected visual acuity (BCVA), central retinal thickness (CRT), number of treatments (anti-vascular endothelial growth factor injections, sub-Tenon triamcinolone acetonide injections, microaneurysm photocoagulation, and pars plana vitrectomy), number of outpatient visits, presence of fibrinogen clot removal, and recurrence were retrospectively analyzed. The mean age was 72.3 ± 10.3 years. Mean BCVA improved from 0.33 ± 0.24 logarithm of the minimum angle of resolution preoperatively to 0.21 ± 0.22 at 3 years (p < 0.001). CRT significantly decreased from 504.7 ± 118.2 μm to 295.2 ± 87.4 μm (p < 0.001). The average annual number of total treatments decreased from 3.7 ± 1.8 to 1.1 ± 3.1 (p < 0.001), and outpatient visits decreased from 11.8 ± 4.0 to 4.4 ± 3.2 (p < 0.001). Fibrinogen clot removal was performed in nine eyes. Recurrence was observed in eight eyes. Cystotomy appeared to be a promising surgical option for managing refractory RVO-ME.

## Introduction

Retinal vein occlusion (RVO) represents a retinal vascular disorder resulting from venous occlusion, frequently associated with systemic conditions such as hypertension and arteriosclerosis. The resultant increase in venous pressure enhances vascular permeability, disrupts the blood-retinal barrier, and induces the formation of microaneurysms (MAs), ultimately leading to retinal hemorrhage and macular edema secondary to RVO (RVO-ME). Impaired retinal circulation due to venous occlusion creates an ischemic and hypoxic environment, which promotes the production of cytokines such as vascular endothelial growth factor (VEGF), leading to microvascular damage and substantial visual impairment [1].

The primary goal of RVO-ME treatment is to control these pathological mechanisms. Currently, a multimodal therapeutic approach combining anti-VEGF therapy, intravitreal or sub-Tenon triamcinolone acetonide injections (STTA), retinal photocoagulation (PC), and pars plana vitrectomy (PPV) is widely employed [1–7]. Although many cases respond to these treatments, some patients continue to exhibit persistent cystoid macular edema (CME), representing a therapeutic challenge [2–4].

Recently, cystotomy—an incision of the inner wall of cystoid spaces—has emerged as a potential option for managing refractory CME [8–9]. A surgical technique developed by our group involves removing fibrinogen clots within the cystoid cavity and has demonstrated favorable short-term visual outcomes [10–14]. However, the long-term efficacy and safety of this procedure remain unclear. Therefore, this study aimed to evaluate the 3-year postoperative outcomes of cystotomy in patients with RVO-ME.

## Materials and methods

### Participants and study design

This retrospective observational study included 23 eyes of 23 patients who underwent cystotomy for RVO-ME at Kobe University Hospital between September 2014 and July 2021, with a follow-up period of at least 3 years. The study was conducted in accordance with the Declaration of Helsinki and approved by the Institutional Review Board of Kobe University Hospital (Approval No. B240036). We accessed the medical records on November 5, 2024, for research purposes and had access to information that could identify individual participants during or after data collection. The requirement for informed consent was waived by the committee because of the retrospective observational design of the study. Nonetheless, patients could opt out and withdraw consent at any time through the hospital homepage.

### Inclusion criteria

Eligible eyes met one of the following criteria: (1) RVO-ME with CME refractory to ranibizumab (0.5 mg) or aflibercept (2 mg) anti-VEGF therapy, STTA, direct laser photocoagulation for microaneurysms (MA-PC), and/or PPV with internal limiting membrane (ILM) peeling; (2) RVO-ME with CME in cases where patients, regardless of treatment history, opted against anti-VEGF or STTA therapy due to frequent visits or financial concerns and preferred surgical intervention via PPV.

### Exclusion criteria

Patients were excluded if they had: (1) cataract graded ≥ grade 3 based on the Emery-Little classification; (2) coexisting glaucoma; (3) a history of uveitis; and (4) previous vitreoretinal surgery for other retinal diseases.

### Data collection and outcome measures

The following data were extracted from medical records: sex, age, lens status (phakic/pseudophakic), best-corrected visual acuity (BCVA), and central retinal thickness (CRT) at baseline and at 1, 3, 6, 12, 24, and 36 months postoperatively. Additional variables included the presence of fibrinogen clot removal [10], recurrence of RVO-ME, the number of treatments (anti-VEGF, STTA, MA-PC, and PPV with ILM peeling) in the year before surgery, and the number of additional treatments at 1, 2, and 3 years postoperatively in recurrent cases. Outpatient visits related to RVO-ME were counted at the same time intervals. CRT was measured using the Cirrus HD-OCT (Carl Zeiss Meditec). Visual acuity was converted to a logarithm of the minimum angle of resolution (logMAR) for statistical analysis. For cases requiring panretinal photocoagulation (PRP), the procedure was performed prior to surgery; therefore, within the observation period for this study, there were cases in which PRP was performed during the year before surgery, but no cases in which PRP was performed after surgery. PRP was not specifically analyzed in this study.

### Surgical procedure

All surgeries were performed under local anesthesia using 2% lidocaine administered via sub-Tenon injection. The Constellation Vision System (Alcon Laboratories, Fort Worth, TX, USA) was used for vitrectomy, and intraocular observation was conducted using a wide-angle non-contact viewing system (Resight^®^; Carl Zeiss Meditec AG, Jena, Germany). In phakic eyes, cataract extraction with intraocular lens implantation was performed before cystotomy. In eyes without prior PPV, a 27-gauge vitrectomy was performed, followed by ILM peeling within an area of approximately two disc diameters centered on the fovea. Subsequently, cystotomy was performed. For cystotomy, the inner wall of the foveal cystoid macular edema was grasped with 27G MaxGrip forceps (Alcon Grieshaber AG, Schaffhausen, Switzerland) and incised radially from the center in a manner similar to peeling the skin of an orange. When a fibrinogen clot was identified within the cystoid cavity [11], it was removed with the same forceps. This procedure was applied only to the fovea and not to other retinal areas. No cases required fluid–air exchange or intraocular gas tamponade at the end of surgery.

### Statistical analysis

Comparisons between the recurrence and non-recurrence groups for age, preoperative BCVA, preoperative CRT, number of treatments, and number of outpatient visits were conducted using unpaired t-tests. Comparisons of categorical variables—sex, lens status, and presence of fibrinogen clot removal—were performed using chi-square tests. To assess longitudinal changes in BCVA, CRT, treatment frequency, and outpatient visit frequency, the Kruskal–Wallis test was applied, followed by Dunn’s post hoc test for multiple comparisons. All statistical analyses were performed using SPSS software (IBM, version 24.0). Statistical significance was set at p < 0.05.

## Results

Preoperative background characteristics of the patients are summarized in Table 1. A total of 23 eyes from 23 patients (10 males and 13 females) were included, with a mean age of 72.3 ± 10.3 years. At the time of surgery, 11 eyes were phakic and 12 were pseudophakic.

**Table 1:** Preoperative background characteristics of the patients.

	Total	Recurrence group	Non-recurrence group	*p*-value
Number of patients	23	8	15	
Sex (male, female)	10, 13	5, 3	5, 10	0.22^†^
Age, average ± SD (years)	72.3 ± 10.3	67.9 ± 12.3	74.7 ± 8.5	0.13^††^
Lens status, phakia/pseudophakia (eyes)	11, 12	4, 4	7, 8	0.78^†^
BRVO/CRVO (eyes)	16, 7	4, 4	12, 3	0.18^†^
Pre BCVA, average ± SD (logMAR)	0.33 ± 0.24	0.37 ± 0.20	0.31 ± 0.25	0.58^††^
Pre CRT, average ± SD (μm)	504.7 ± 118.2	546.1 ± 117.0	482.5 ± 112.8	0.29^††^
Fibrinogen clot removal (yes/no)	9, 14	3, 5	6, 9	0.95^†^
Number of total treatments in the year before surgery (average ± SD)	3.7 ± 1.7	4.0 ± 2.1	3.8 ± 1.3	0.33^††^
Anti-VEGF therapy	2.6 ± 1.6	3.0 ± 1.4	2.6 ± 1.1	0.28^††^
STTA	0.3 ± 0.8	0.1 ± 0.3	0.5 ± 0.9	0.16^††^
MA-PC	0.6 ± 0.8	0.8 ± 1.0	0.6 ± 0.9	0.38^††^
PPV	0.1 ± 0.3	0.1 ± 0.3	0.1 ± 0.3	0.49^††^
Number of visits in the year before surgery (average ± SD)	11.5 ± 4.3	13.6 ± 3.0	10.4 ± 4.5	0.12^††^

BCVA, best-corrected visual acuity; CMT, central macular thickness; MA-PC, photocoagulation for microaneurysm; PPV, pars plana vitrectomy; STTA, sub-Tenon triamcinolone acetonide administration; VEGF, vascular endothelial growth factor; SD, standard deviation.

† : chi-square test,

†† : unpaired t-test.

**Fig 1:**
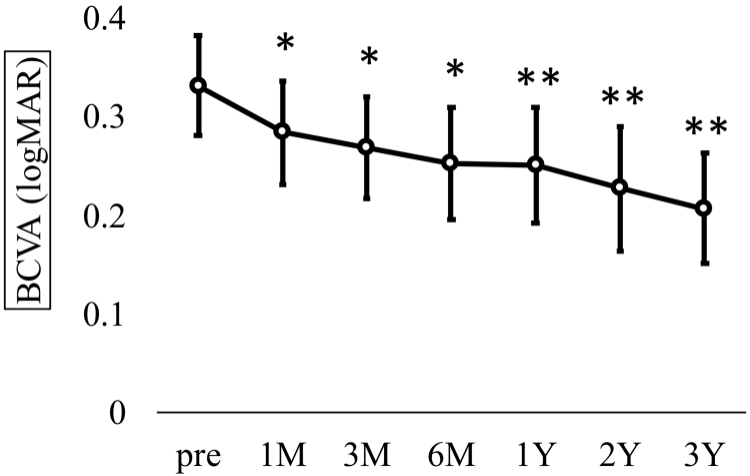
Time course of best-corrected visual acuity (BCVA) in all eyes. Mean BCVA (logMAR ± SD) improved significantly from 0.33 ± 0.24 preoperatively to 0.28 ± 0.25 at 1 month, 0.27 ± 0.24 at 3 months, 0.25 ± 0.26 at 6 months, 0.25 ± 0.28 at 12 months, 0.23 ± 0.30 at 24 months, and 0.21 ± 0.22 at 36 months postoperatively (Kruskal–Wallis test, P < 0.001; Dunn’s test, *P < 0.05, **P < 0.01). CRT also showed a significant reduction from 504.7 ± 118.2 μm preoperatively to 302.5 ± 60.5 at 1 month, 307.5 ± 64.4 at 3 months, 286.8 ± 49.0 at 6 months, 305.3 ± 79.5 at 12 months, 288.8 ± 79.1 at 24 months, and 295.2 ± 87.4 at 36 months (Kruskal-Wallis test, p < 0.001; Fig 2).

In the overall analysis, BCVA (logMAR) significantly improved from 0.33 ± 0.24 preoperatively to 0.28 ± 0.25 at 1 month, 0.27 ± 0.24 at 3 months, 0.25 ± 0.26 at 6 months, 0.25 ± 0.28 at 12 months, 0.23 ± 0.30 at 24 months, and 0.21 ± 0.22 at 36 months postoperatively (Kruskal-Wallis test, p < 0.001; Fig 1).

**Fig 2:**
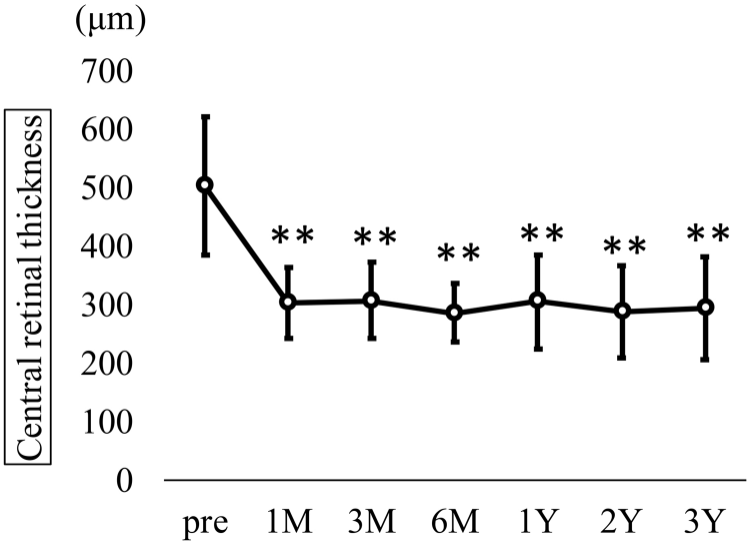
Time course of central retinal thickness (CRT) in all eyes. Mean CRT (μm ± standard deviation) significantly decreased from 504.7 ± 118.2 μm preoperatively to 302.5 ± 60.5 at 1 month, 307.5 ± 64.4 at 3 months, 286.8 ± 49.0 at 6 months, 305.3 ± 79.5 at 12 months, 288.8 ± 79.1 at 24 months, and 295.2 ± 87.4 at 36 months (Kruskal–Wallis test, P < 0.001; Dunn’s test, **P < 0.01). Fibrinogen clots were removed intraoperatively in nine out of 23 eyes (39.1%). The mean number of treatments—including anti-VEGF, STTA, MA-PC, and PPV—in the year before surgery was 2.6 ± 1.6, 0.3 ± 0.8, 0.6 ± 0.8, and 0.1 ± 0.3, respectively, with a total of 3.7 ± 1.7 treatments. These treatment frequencies declined significantly after surgery: at 1 year postoperatively, the averages were 0.8 ± 1.9, 0.0 ± 0.2, 0.3 ± 1.0, 0.0, and 1.2 ± 2.2; at 2 years, 0.7 ± 2.0, 0.1 ± 0.6, 0.0 ± 0.2, 0.0 ± 0.2, and 1.0 ± 2.1; and at 3 years, 0.9 ± 2.2, 0.0, 0.2 ± 1.0, 0.0 ± 0.2, and 1.1 ± 3.1 (Kruskal-Wallis test, p < 0.001; Fig 3). All PPVs performed after cystotomy were repeat cystotomies in eyes with recurrence.

**Fig 3:**
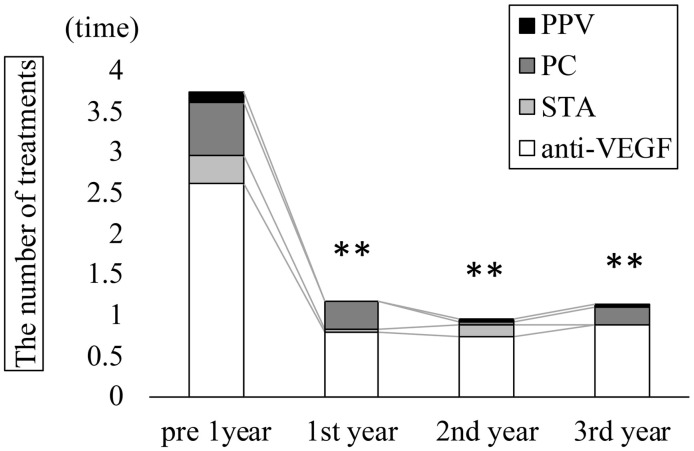
Annual treatment frequencies in all eyes. The number of anti-VEGF treatments, STTA, MA-PC, PPV, and total treatments (mean ± SD) significantly decreased from 2.6 ± 1.6, 0.3 ± 0.8, 0.6 ± 0.8, 0.1 ± 0.3, and 3.7 ± 1.7 preoperatively to 0.8 ± 1.9, 0.0 ± 0.2, 0.3 ± 1.0, 0.0, and 1.2 ± 2.2; at year 2 to 0.7 ± 2.0, 0.1 ± 0.6, 0.0 ± 0.2, 0.0 ± 0.2, and 1.0 ± 2.1; and at year 3 to 0.9 ± 2.2, 0.0, 0.2 ± 1.0, 0.0 ± 0.2, and 1.1 ± 3.1 (Kruskal–Wallis test, P < 0.001; Dunn’s test, **P < 0.01). VEGF: vascular endothelial growth factor; STTA: sub-Tenon triamcinolone acetonide; PC: photocoagulation; PPV: pars plana vitrectomy. Outpatient visit frequency also significantly declined from 11.5 ± 4.3 visits in the year before surgery to 8.8 ± 4.1, 5.0 ± 3.4, and 4.4 ± 3.2 visits in the first, second, and third postoperative years, respectively (Kruskal-Wallis test, p < 0.001; Fig 4).

**Fig 4:**
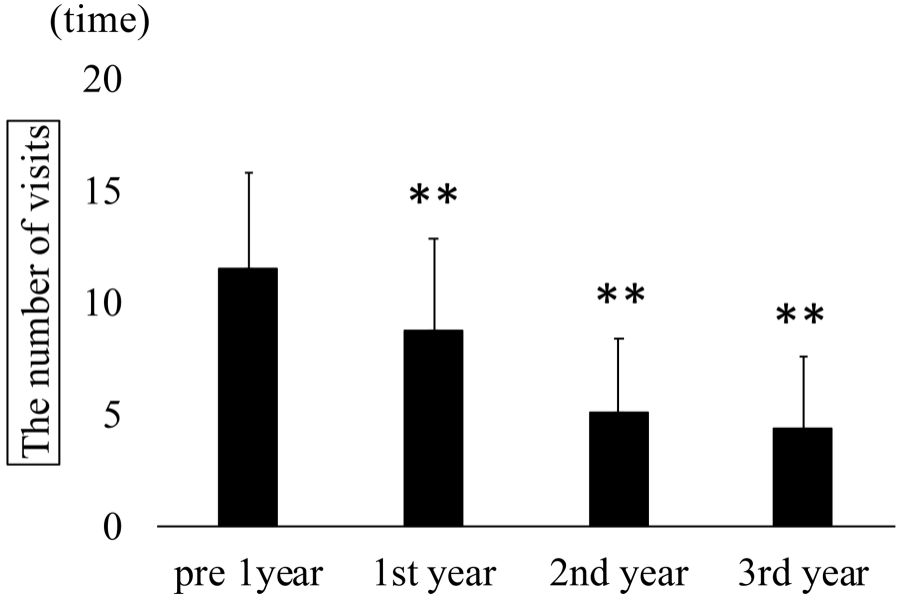
Annual number of outpatient visits in all eyes. Mean visit frequency (mean ± standard deviation) significantly decreased from 11.5 ± 4.3 preoperatively to 8.8 ± 4.1, 5.0 ± 3.4, and 4.4 ± 3.2 visits in the first, second, and third postoperative years, respectively (Kruskal–Wallis test, P < 0.001; Dunn’s test, **P < 0.01). Recurrence was defined as the presence of intraretinal or subretinal fluid at the fovea on optical coherence tomograpy (OCT), that is, the reappearance of CME. Additional treatments were administered as needed. Recurrence occurred in eight eyes (34.8%), whereas 15 eyes (65.2%) had no recurrence. No significant differences were found between the recurrence and non-recurrence groups regarding sex, age, lens status, type of RVO (BRVO or CRVO), preoperative BCVA and CRT, presence of fibrinogen clot removal, or the number of treatments and visits during the year before surgery (Table 1). In the recurrence group, BCVA (logMAR) showed no significant improvement over time: 0.37 ± 0.20 preoperatively and 0.35 ± 0.23, 0.35 ± 0.18, 0.31 ± 0.23, 0.34 ± 0.26, 0.28 ± 0.30, and 0.24 ± 0.23 at 1, 3, 6, 12, 24, and 36 months, respectively (Kruskal-Wallis test, p = 0.45). In contrast, the non-recurrence group demonstrated significant and sustained improvement from 0.31 ± 0.25 preoperatively to 0.25 ± 0.25, 0.23 ± 0.26, 0.23 ± 0.28, 0.21 ± 0.27, 0.20 ± 0.30, and 0.16 ± 0.30 at corresponding postoperative time points (Kruskal-Wallis test, p < 0.001; Fig 5).

**Fig 5:**
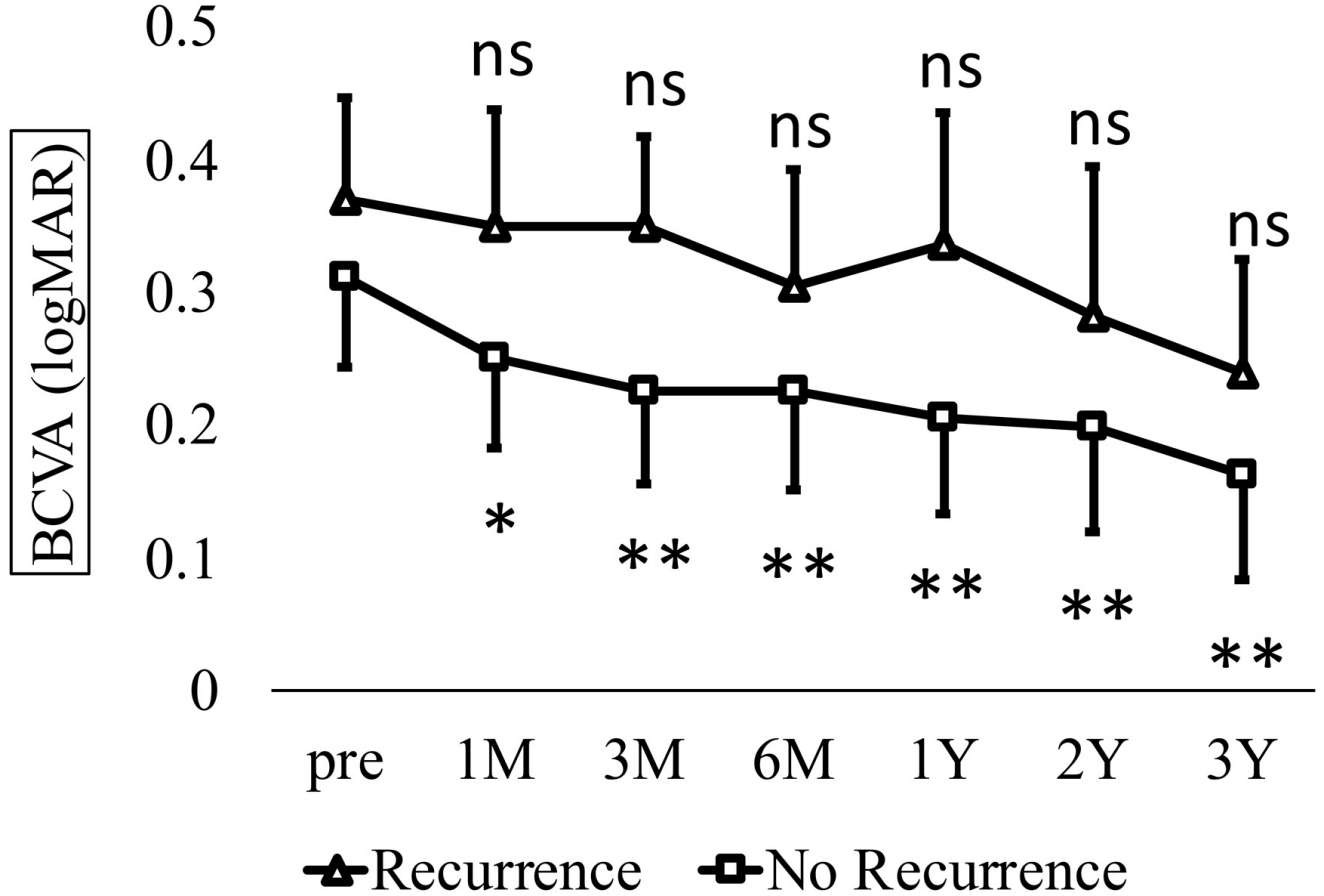
Time course of best-corrected visual acuity (BCVA) in recurrence and non-recurrence groups. In the recurrence group, BCVA (logMAR ± standard deviation) showed no significant change from baselines (0.37 ± 0.20) to 0.35 ± 0.23, 0.35 ± 0.18, 0.31 ± 0.23, 0.34 ± 0.26, 0.28 ± 0.30, and 0.24 ± 0.23 at 1, 3, 6, 12, 24, and 36 months, respectively (Kruskal–Wallis test, P = 0.45). In contrast, BCVA in the non-recurrence group significantly improved from 0.31 ± 0.25 preoperatively to 0.25 ± 0.25, 0.23 ± 0.26, 0.23 ± 0.28, 0.21 ± 0.27, 0.20 ± 0.30, and 0.16 ± 0.30 over the same time points (Kruskal–Wallis test, P < 0.001; Dunn’s test, *P < 0.05, **P < 0.01). CRT in the recurrence group significantly decreased from 546.1 ± 117.0 μm preoperatively to 317.1 ± 46.9, 317.8 ± 55.6, 290.8 ± 33.5, 342.9 ± 82.2, 333.0 ± 84.2, and 349.8 ± 76.9 μm at 1, 3, 6, 12, 24, and 36 months postoperatively, respectively (Kruskal-Wallis test, p < 0.001). In the non-recurrence group, CRT improved from 482.5 ± 112.8 to 294.7 ± 65.3, 302.0 ± 68.0, 284.7 ± 55.4, 285.3 ± 70.2, 265.2 ± 65.0, and 266.1 ± 78.2 μm at 1, 3, 6, 12, 24, and 36 months, respectively (Kruskal-Wallis test, p < 0.001; Fig 6).

**Fig 6:**
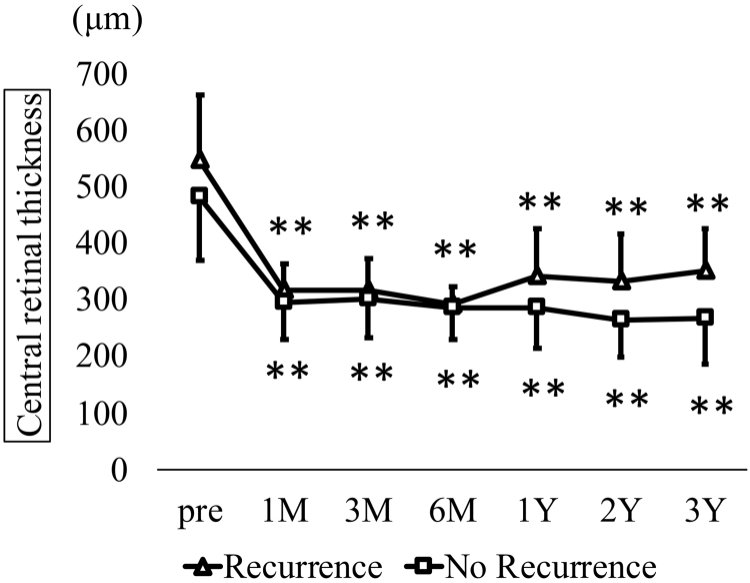
Time course of central retinal thickness (CRT) in recurrence and non-recurrence groups. In the recurrence group, CRT (μm ± standard deviation) significantly decreased from 546.1 ± 117.0 μm preoperatively to 317.1 ± 46.9, 317.8 ± 55.6, 290.8 ± 33.5, 342.9 ± 82.2, 333.0 ± 84.2, and 349.8 ± 76.9 μm at 1, 3, 6, 12, 24, and 36 months, respectively (Kruskal-Wallis test, P < 0.001; Dunn’s test, **P < 0.01). In the non-recurrence group, CRT improved from 482.5 ± 112.8 to 294.7 ± 65.3, 302.0 ± 68.0, 284.7 ± 55.4, 285.3 ± 70.2, 265.2 ± 65.0, and 266.1 ± 78.2 μm at 1, 3, 6, 12, 24, and 36 months, respectively (Kruskal–Wallis test, P < 0.001; Dunn’s test, **P < 0.01). Regarding the mean number of treatments in the recurrence group, anti-VEGF, STTA, MA-PC, PPV, and total treatment counts in the year before surgery were 3.0 ± 1.4, 0.1 ± 0.3, 0.8 ± 1.0, 0.1 ± 0.3, and 4.0 ± 2.1, respectively. These values significantly decreased in the first postoperative year to 2.3 ± 2.6, 0.1 ± 0.3, 0.8 ± 1.6, 0, and 3.1 ± 2.8; in the second year to 2.1 ± 2.8, 0.4 ± 1.0, 0.0, 0.1 ± 0.3, and 2.6 ± 2.8; and in the third year to 2.0 ± 2.2, 0, 0.6 ± 1.7, 0.1 ± 0.3, and 2.8 ± 3.5 (Kruskal–Wallis test, p < 0.001; Fig 7). All PPVs performed after cystotomy were repeat cystotomies in eyes with recurrence.

**Fig 7:**
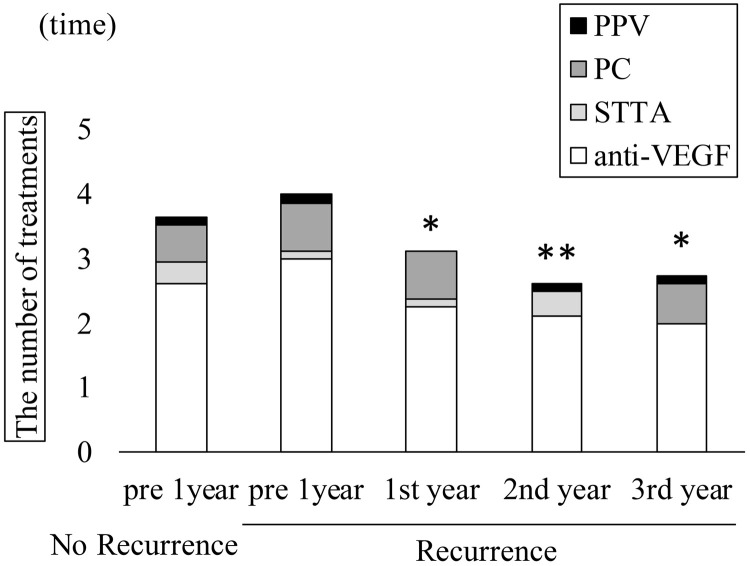
Annual treatment frequencies in recurrence and non-recurrence groups. In the recurrence group, the respective treatment counts for anti-VEGF, STTA, MA-PC, PPV, and total treatments (mean ± standard deviation) in the year before surgery were 3.0 ± 1.4, 0.1 ± 0.3, 0.8 ± 1.0, 0.1 ± 0.3, and 4.0 ± 2.1, respectively. These significantly decreased to 2.3 ± 2.6, 0.1 ± 0.3, 0.8 ± 1.6, 0.0, and 3.1 ± 2.8 in the first year; 2.1 ± 2.8, 0.4 ± 1.0, 0, 0.1 ± 0.3, and 2.6 ± 2.8 in the second year; and 2.0 ± 2.2, 0, 0.6 ± 1.7, 0.1 ± 0.3, and 2.8 ± 3.5 in the third year (Kruskal–Wallis test, p < 0.001; Dunn’s test, *P < 0.05, **P < 0.01). In the non-recurrence group, the respective preoperative treatment counts were 2.6 ± 1.1, 0.5 ± 0.9, 0.6 ± 0.9, 0.1 ± 0.3, and 3.8 ± 1.3, and no additional treatments were required during the 3 postoperative years. VEGF: vascular endothelial growth factor; STTA: sub-Tenon triamcinolone acetonide; PC: photocoagulation; PPV: pars plana vitrectomy. In the non-recurrence group, the respective preoperative treatment counts were 2.6 ± 1.1, 0.5 ± 0.9, 0.6 ± 0.9, 0.1 ± 0.3, and 3.8 ± 1.3, and no additional treatments were required during the 3 postoperative years. (Fig 7). Mean outpatient visits in the recurrence group decreased from 13.6 ± 3.0 to 11.9 ± 5.0, 8.1 ± 3.9, and 7.8 ± 3.2 at 1, 2, and 3 years postoperatively, respectively (Kruskal-Wallis test, p < 0.001). In the non-recurrence group, visits declined from 10.4 ± 4.5 preoperatively to 7.1 ± 2.2, 3.4 ± 1.3, and 2.6 ± 1.0 at 1, 2, and 3 years postoperatively, respectively (Kruskal-Wallis test, p < 0.001; Fig 8).

**Fig 8:**
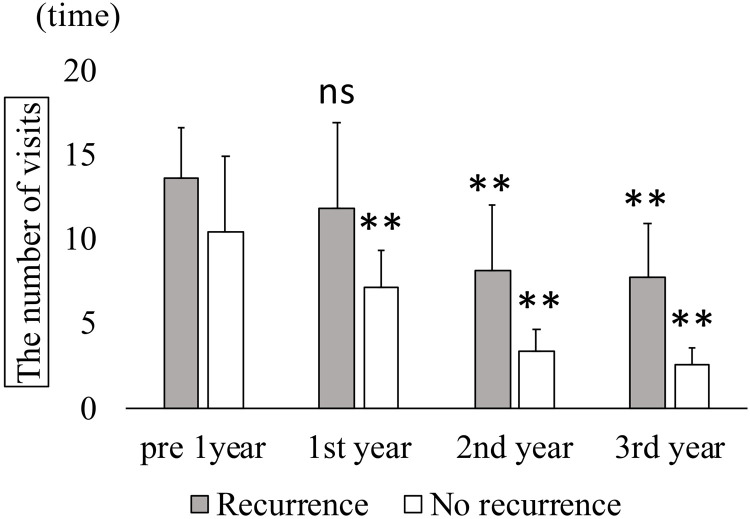
Annual number of outpatient visits in recurrence and non-recurrence groups. In the recurrence group, mean outpatient visits (± standard deviation) decreased from 13.6 ± 3.0 to 11.9 ± 5.0, 8.1 ± 3.9, and 7.8 ± 3.2 at 1, 2, and 3 years postoperatively, respectively (Kruskal-Wallis test, P < 0.001; Dunn’s test, **P < 0.01). In the non-recurrence group, visits declined from 10.4 ± 4.5 preoperatively to 7.1 ± 2.2, 3.4 ± 1.3, and 2.6 ± 1.0 at 1, 2, and 3 years postoperatively, respectively (Kruskal–Wallis test, P < 0.001; Dunn’s test, **P < 0.01). Fig 9 presents the time course of OCT and fundus findings in a representative non-recurrent case. A 73-year-old man with RVO-ME in the left eye previously underwent eight anti-VEGF injections, two sessions of MA-PC, and PPV with ILM peeling without achieving improvement. Cystotomy was subsequently performed. Preoperative BCVA measured 0.4, and OCT revealed RVO-ME **(A)**. At 3 months postoperatively, the edema had resolved **(C)**, and BCVA improved to 0.6. No recurrence of RVO-ME was observed during follow-ups at 6 months **(E)**, 1 year **(G)**, and 3 years (I) postoperatively. BCVA further improved to 0.7 at 3 years. Fundus photographs showed no evidence of foveal chorioretinal atrophy related to the surgical procedure during follow-up (B: preoperative, D: 3 months, F: 6 months, H: 1 year, and J: 3 years).

**Fig 9:**
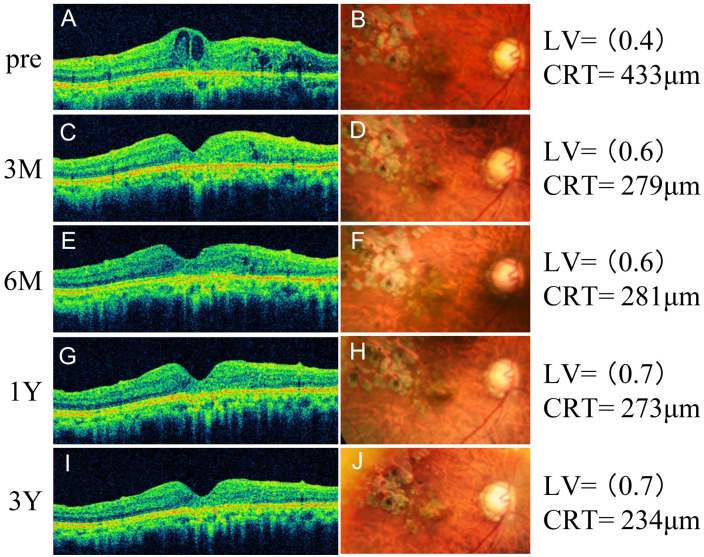
Time course of optical coherence tomography and fundus findings in a representative non-recurrent eye. A 73-year-old man with RVO-ME in the left eye had previously received eight anti-VEGF injections, two sessions of MA-PC, and PPV with ILM peeling without achieving improvement. Cystotomy was subsequently performed. Preoperative BCVA measured 0.4, and OCT revealed RVO-ME **(A)**. At 3 months postoperatively, the edema had resolved **(C)**, and BCVA improved to 0.6. No recurrence of RVO-ME was observed at 6 months **(E)**, 1 year **(G)**, and 3 years **(I)** postoperatively. BCVA further improved to 0.7 at 3 years. Fundus photographs showed no signs of foveal chorioretinal atrophy related to the surgical procedure during follow-up (**B:** preoperative, **D:** 3 months, **F:** 6 months, **H:** 1 year, and **J:** 3 years). VEGF: vascular endothelial growth factor, MA-PC: photocoagulation for microaneurysm, PPV: pars plana vitrectomy, DME: diabetic macular edema, OCT: optical coherence tomography.

## Discussion

In recent years, cystotomy has garnered attention as a novel surgical approach for treating CME. Several studies have reported its efficacy across various types of refractory CME [8–14]. The initial report on cystotomy involved 22 eyes with diabetic macular edema (DME), where the procedure was incorporated into primary vitrectomy without ILM peeling. Edema resolution was achieved in 72.7% (16/22 eyes), and visual acuity either improved or remained stable in 90% (20/22 eyes) [8]. Despite differences in pathogenesis between RVO-ME and DME [15,16], both conditions share common pathological features, including Müller cell junction dysfunction and disruption of the perifoveal capillary barrier. These changes contribute to fluid accumulation within the Henle fiber layer and the formation of cystoid macular edema [17]. Previously, we reported the potential utility of cystotomy with fibrinogen clot removal for managing RVO-ME [12]. This study, which included extended follow-up, demonstrated significant improvements in both BCVA and CRT within 1 month postoperatively, with sustained benefits maintained for up to 36 months. These findings suggest that cystotomy provides durable therapeutic effects in cases of treatment-resistant RVO-ME, including those unresponsive to standard interventions.

Currently, anti-VEGF therapy is considered the first-line treatment for RVO-ME. Multiple large-scale prospective studies have confirmed superior visual outcomes with anti-VEGF agents compared to corticosteroids or laser photocoagulation [2–4,18,19], and real-world data have further substantiated these findings [20–23]. However, studies have reported that an average of 5.4 to 6.0 injections per year may remain necessary even beyond the second year of therapy [22,23], underscoring the continued demand for frequent clinical visits and treatments to preserve or improve visual function. In contrast, this study demonstrated a significant reduction in both treatment and visit frequency following cystotomy. Notably, no additional treatment was required over the 3-year follow-up in the non-recurrence group, suggesting that cystotomy may substantially alleviate both physical and financial burdens on patients. Recurrence of RVO-ME occurred in eight out of 23 eyes (34.8%), and the recurrence group did not exhibit significant visual improvement. The recent availability of faricimab in Japan has introduced the possibility of extending injection intervals to 12–16 weeks [4]. With such advances in anti-VEGF therapy, caution should be exercised when considering surgical intervention as a first-line option. Nevertheless, in this study, 15 eyes (65.2%) showed no recurrence and demonstrated significant visual improvement over 3 years, indicating that cystotomy may offer favorable outcomes in selected patients. Although this study did not identify specific predictive factors for recurrence, further investigation may enable the identification of suitable candidates who would benefit most from this approach. If such criteria can be established, cystotomy could become a valuable addition to the therapeutic options available for RVO-ME. Moreover, for patients who exhibit poor response to conventional therapies or face difficulties in adhering to frequent treatment regimens, cystotomy may already serve as a meaningful and practical alternative.

This study had several limitations. Firstly, as a retrospective uncontrolled observational study, it was inherently limited in its ability to establish causal relationships. Secondly, the relatively small sample size limited the generalizability of the findings. Future prospective studies with larger cohorts are needed to enhance the reproducibility and reliability of the results. Furthermore, extending the follow-up period would allow for a more comprehensive assessment of the long-term efficacy and safety of the procedure.

## Conclusions

In conclusion, the findings of this study suggested that cystotomy may serve as an effective therapeutic option for RVO-ME. With further clarification of recurrence risk factors and refinement of appropriate indications, this procedure has the potential to become an established treatment strategy that improves quality of life and reduces treatment burden for affected patients.
